# Managing Pain in Cerebral Palsy Patients Using Ethanol Hip Joint Injection: A Retrospective Chart Review

**DOI:** 10.7759/cureus.90603

**Published:** 2025-08-20

**Authors:** Shea I Buckley, Sterling C Kneedler, David A Yngve

**Affiliations:** 1 Department of Orthopaedic Surgery and Rehabilitation, University of Texas Medical Branch, Galveston, USA

**Keywords:** cerebral palsy, ethanol, hip dysplasia, injections, nonoperative treatment, pain management, treatment effectiveness

## Abstract

Background

In people with cerebral palsy (CP), some painful hips are too dysplastic for reconstruction by femoral or acetabular osteotomies. For these hips, a less invasive treatment than a bone-removing salvage operation is needed. The nonoperative management of this pain is not well-studied. The purpose of this study is to evaluate the use of ethanol hip joint injection to alleviate hip pain. This study aims to evaluate the outcomes of ethanol hip joint injections administered by a single surgeon and to evaluate their effectiveness in alleviating hip joint pain.

Methods

We included 57 consecutive patients with 71 hips in this study, with moderate-to-severe or severe pain. They had a total of 104 injections. Injections consisted of 10 mL to 15 mL of 75% ethanol into the hip joint under C-arm control, using a sterile field and general anesthesia. Gross Motor Function Classification System (GMFCS) was 4 or 5 for all. Data was collected from January 2010 to March 2023. The primary outcome was hip pain as recorded in the medical record. This was based on caregivers’ perceptions and patients’ facial expressions during physical exams in the clinic. Pain was evaluated on a scale of 1 to 5 (1: mild, 2: mild-moderate, 3: moderate, 4: moderate to severe, 5: severe). Following injection, a reduction in pain to a level less than 4 was considered a clinical success. The average follow-up period for these patients was 43 months (0.5-144 months). Short follow-up times gave information on the early onset, while longer follow-up times gave information on the duration of action. The total number of follow-up clinic visits for these patients was 202, and each clinic visit was used for pain score and follow-up time. A time-to-event analysis was done using Kaplan-Meier cumulative event curves to evaluate (1) time to a self-reported subjective pain score below 4 following the first injection and (2) to estimate the duration of pain control after the first injection. The event of interest was the time to the second injection in a patient who had reported a pain score below 4 after their first injection.

Results

The average patient age was 16 years. Of the 71 hips treated with a first ethanol hip joint injection, 61 (86%) showed a decrease in pain score to below 4. The median time to documentation of pain control after a first injection was four months. Within 10 months, 85% of the patients' hips reported pain control. Of those 61 hips with a decrease in pain score to below 4, 19 (31%) required a second injection. The median time to second injection after achieving pain control with the first injection was 33 months. When looking at all 104 injections, 36% experienced pain relapse. In our population who had hip joint injections, 11 hips (15%) had salvage surgeries and six hips (8%) developed avascular necrosis of the femoral head as an incidental X-ray finding.

Conclusion

This is the first study of an important modality. For our population of patients with CP and GMFCS level of 4 or 5, an injection of 10 mL to 15 mL of 75% ethanol into the affected hip joint, along with other procedures, provided pain control in 86%, with 31% of patients having a second injection after a median of 33 months. Based on these results, ethanol injection into the hip joint in those with severe or moderately severe pain should be further studied and considered as a treatment modality.

## Introduction

Nonambulatory people with cerebral palsy (CP) often experience severe hip pain due to an interplay of spasticity, increased muscle tone, contractures, and hip dysplasia. This pain significantly impacts their daily life and well-being. Current nonoperative pain management strategies are often insufficient, leading many patients and families to seek surgical options. Nonoperative treatments include botulinum toxin injections, intrathecal baclofen, oral gabapentin, massage, and dorsal root ganglion radiofrequency ablation [1,2]. When these treatments fail, surgical options to decrease painful pressure between the proximal femur and the pelvis, such as the modified McHale procedure, Castle procedure, and Girdlestone procedure, are available [1,2].

Throughout history, the use of ethanol as a modality for pain control has been studied at the basic science level in both animal models and human studies. However, few controlled studies exist; thus, the literature primarily consists of observations, reports, and book chapters reflecting the opinions of experts and experienced clinicians.

Mechanistically, ethanol acts to demyelinate axons by damaging Schwann cells through a dehydration event, leading to the extraction of cholesterol, phospholipids, and cerebrosides, which precipitate mucoproteins. This process can lead to Wallerian degeneration depending on the dose [3-5]. In vivo electrophysiologic investigations of peripheral nerves in cats revealed significantly depressed compound action potentials (CAP) of large A, A-delta, and C fibers when tested eight weeks after ethanol injection of 0.5 mL near the nerve. There was a small increase in effect on CAP as ethanol concentration increased from 50% to 100%; however, the 100% solution caused marked skin sloughing [6].

Ethanol and phenol were commonly used for intrathecal neurolytic blocks in chronic pain management, targeting the dorsal root ganglia to preserve motor function while eliminating pain sensation [5]. Staats et al. [7,8] evaluated the effect of ethanol block in patients with histologically proven, unresectable pancreatic cancer versus saline block (placebo) on pain, mood, interference of pain with activities, and longevity. Their technique involved injecting 20 cc of 50% ethanol directly onto the bilateral celiac ganglia, totaling 40 cc. Their results showed that ethanol intervention had a significant positive effect on life duration and mood scores compared to medical management alone.

El-Sayed [9] conducted a study including 20 patients with severe, intractable pain not relieved by intravenous/oral medications. They injected 100% ethanol for subarachnoid neurolytic block (dorsal rhizotomy). Visual analog scale (VAS) scores for pain, patient satisfaction, and complications were recorded at 24 hours, one week, and one, two, and three months after the procedure. VAS scores significantly decreased, and patient satisfaction significantly increased at the measured time points. Candido and Stevens [5], in a study on intrathecal neurolytic blocks for the relief of cancer pain, further summarized the utility of ethanol blockade in chronic pain treatment.

Ethanol has also been used in the treatment of spasticity. Kocabas et al. [10] conducted a study with 20 patients who had sustained a hemiplegic stroke. They were randomized to receive a single treatment of ethanol or phenol targeting the motor branches of the tibial nerve. A nerve stimulator was used to identify the nerve, upon which 5 cc of 50% ethanol was injected. The results showed a significant reduction in ankle plantar flexion in all 10 patients who received ethanol. The effect was seen immediately after the motor branch block and was maintained over a six-month follow-up period in nine patients.

Ethanol injection is not without risks. Patients can develop neuritis, paresis, or paralysis of the targeted nerve [5,11,12]. Additionally, Chacha et al. [13] described the effects of three repeated intra-articular injections of 70% ethanol, a moderately high concentration. Three repeated injections will cause more damage than a single injection. The injections were given on days one, four, and seven in rabbit knee joints. When the animals were sacrificed 24 weeks later, they exhibited joint space narrowing, subchondral sclerosis, and osteophyte formation. Histological review showed extensive cartilage cell death. Caston et al. [14] theorized that the development of arthritis could be attributed to the neurolysis and destruction of sensory innervation to the synovium and joint capsule. These studies suggest that with repeated doses of a high concentration of ethanol within one week, damage is increased. In the current study on ethanol hip joint injections in people with CP, injections were single injections and, if repeated, about a year later.

There is a lack of high-quality research on the efficacy of pain management in these patients within the current literature. Consequently, exploring alternative nonoperative methods is crucial to minimize or delay the need for surgery. This study aims to evaluate the outcomes of ethanol hip joint injections administered by a single surgeon and to evaluate their effectiveness in alleviating hip joint pain.

## Materials and methods

This study received approval from our institution’s Institutional Review Board (study approval #07-166), citing "Written documentation of consent is waived in accordance with 45 CFR 46.117(c)." Our study is a retrospective case series with a quantitative approach. Appropriate consent was obtained.

Study population

This retrospective chart review study included all consecutive patients with nonambulatory CP and a Gross Motor Function Classification System (GMFCS) level of 4 or 5 who had an initial ethanol hip joint injection for pain that was moderate to severe or severe and at least one follow-up clinic visit. The average age at the time of injection was 16 years (Table 1). Data was collected from January 2010 to March 2023. We included 57 unique patients and 71 unique patient hips, totaling 104 injections. The average follow-up period was 43 months (range, 0.5 to 144 months). Short follow-up times provided information on early onset results from the injections, while longer follow-up times gave information on the duration of action. The total number of follow-up clinic visits for these patients was 202, and each clinic visit was used for pain score and follow-up time data.

**Table 1:** Descriptive statistics of study population. IQR, interquartile range; SD, standard deviation

Variable	Mean (SD)	Median (IQR)	Min/Max
Injections per patient hip	1.47 (0.67)	1 (1, 2)	1/4
Age in years, per patient hip injection	16.2 (8.09)	15 (10, 19)	3/54

Study measures

Patient characteristics such as age, sex, acetabular index, femoral head uncovered percentage, and hip abduction value (degrees) at the preoperative visit and GMFCS were recorded. The primary outcome was hip pain, evaluated from the clinic visit chart reviews. The measurement of pain control was based on caregivers’ perceptions and patients’ facial expressions during physical exams in the clinic. Pain was rated on a scale from 1 to 5 (mild, mild-moderate, moderate, moderate-severe, and severe). All patients had pain scores of 4 or 5 prior to their injections. A reduction in the pain scale to less than 4 following injection was considered a clinical success. The presence of avascular necrosis of the femoral head was noted by review of initial and follow-up X-rays.

Injection procedure

**Figure 1:**
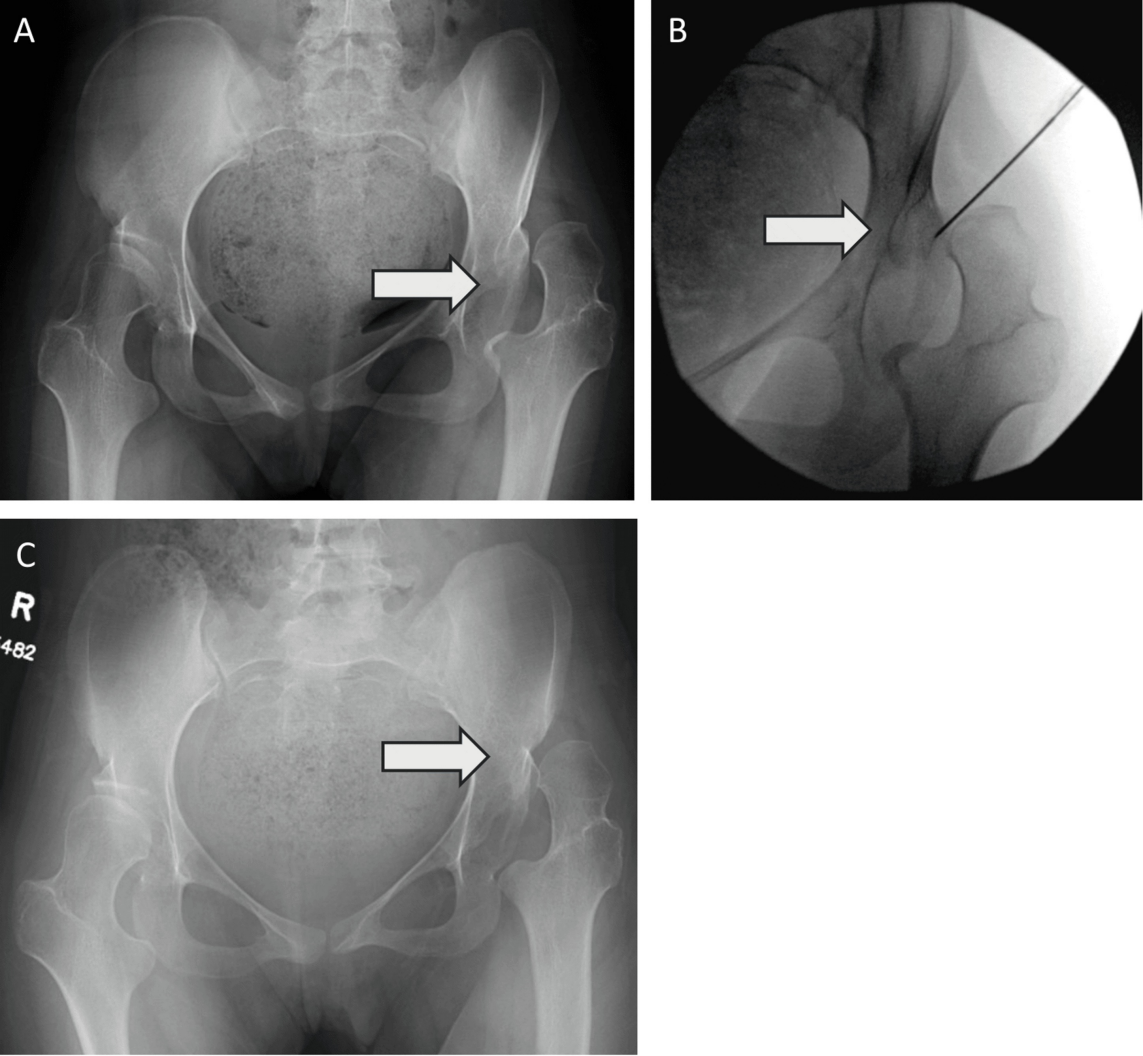
Case requiring one hip joint injection procedure. (A) A 17-year-old female with CP presented with severe left hip pain. (B) She underwent left hip joint injection, with 10 mL of 75% ethanol, bilateral percutaneous adductor release, bilateral obturator nerve block with 3 mL of 50% ethanol, and bilateral percutaneous release of four hamstrings. She returned for five follow-up visits from three to 45 months after the injection, with no left hip pain at any of those visits. (C) Follow-up at 45 months. CP, cerebral palsy

Patients were taken to the operating room (OR) and anesthetized with general anesthesia. Positioned supine, they were sterilely prepped and draped. A C-arm X-ray image intensifier was used. A #17 Tuohy needle was placed into the hip capsule from a direct anterior approach (Figure 1). Once the position was confirmed, 10 mL to 15 mL of 75% ethanol was injected. The injection should proceed with no resistance to the syringe if the needle is properly placed into the joint cavity.

Statistical analysis

Time-to-event analysis was performed using Kaplan-Meier cumulative event curves to evaluate the following: (1) time to a self-reported pain score below 4 and (2) duration of pain control following the first injection. For the first part of the survival analysis, curves were constructed for the time to pain control after the first injection and for all subsequent injections. The event of interest was a self-reported subjective pain score of below 4. For the second part, the event of interest was the time to the second injection for patients who reported a pain score below 4 after their first injection.

Fisher’s exact test was used to look for any relationship between avascular necrosis and efficacious pain management after the first ethanol injection. All analysis was completed using RStudio.

## Results

In this study, pre-injection characteristics were a mean hip abduction range of motion of -0.6 degrees (range, -35 to 35 degrees). Note: minus abduction is equivalent to positive adduction. Additionally, a mean acetabular index of 33 degrees (range, 10-90 degrees), and a mean femoral head uncovered of 75% (range, 0-100%).

**Figure 2:**
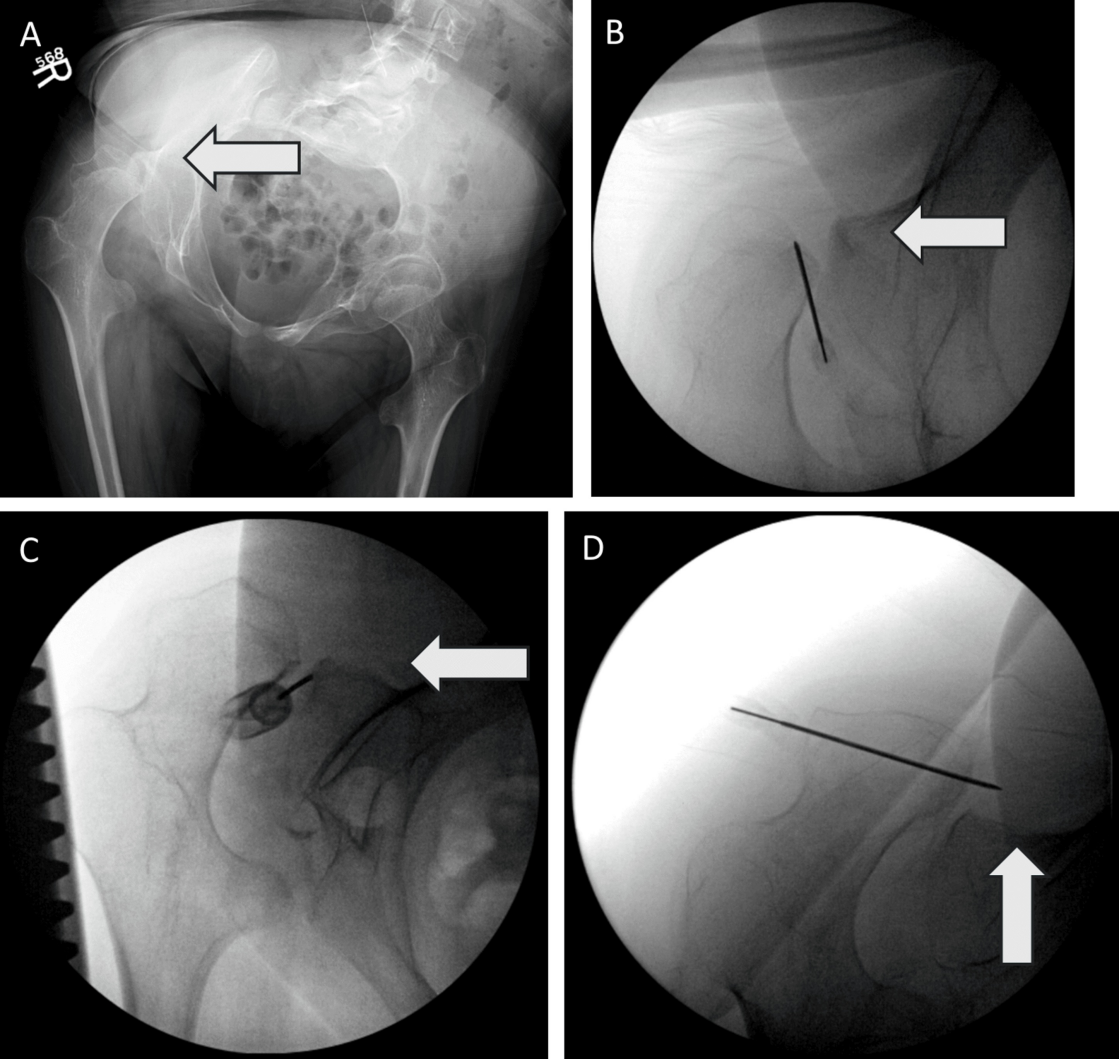
Case requiring three hip joint injection procedures. (A) A 19-year-old male with CP presented with severe right hip pain. (B) He underwent right hip joint injection, with 10 mL of 75% ethanol, bilateral percutaneous adductor release, and bilateral obturator nerve block with 3 mL of 50% ethanol. He returned with his family 18 months after the injection with moderate-to-severe left hip pain, which had returned 11 months after the injection. (C) At 19 months after the first injection, the patient underwent a second right hip joint injection with 15 mL of 75% ethanol and right percutaneous adductor release, and right obturator nerve block with 3 mL of 50% ethanol. He returned for a follow-up two months later with minimal left hip pain. (D) At 17 months after the second injection, the patient returned with severe right hip pain and underwent a third right hip joint injection with 15 mL of 75% ethanol, right percutaneous adductor release, and right obturator nerve block with 3 mL of 50% ethanol. He returned for a follow-up five months later with no right hip pain. CP, cerebral palsy

**Figure 3:**
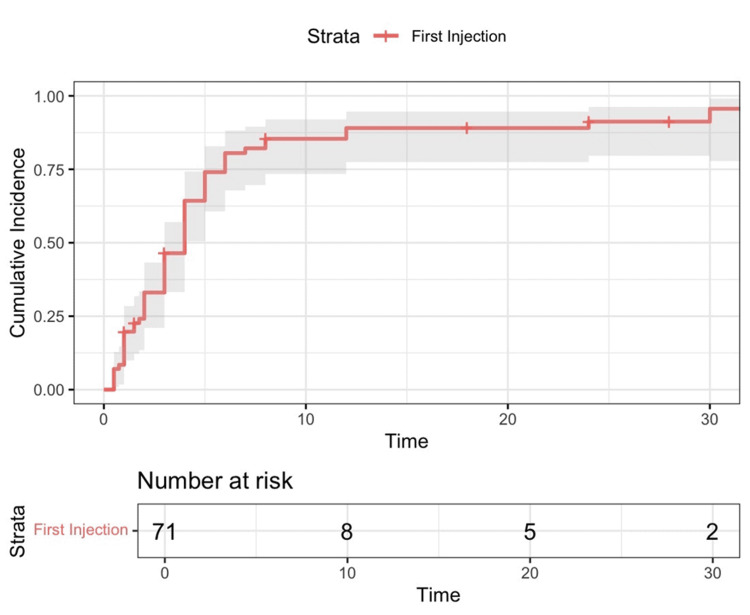
Kaplan-Meier cumulative incidence curve for first injection. Time is in months. A cumulative incidence of 1.00 represents 71 hips. Of the 71 patient hips treated with a first ethanol injection, 61 showed a decrease in pain score to below 4. The median time to pain control after the first injection was four months. The survival rate at 10 months was 0.146, meaning 85% ((1-0.146)×100) of the patient hips reported pain control within 10 months.

Of the 71 hips treated with the first ethanol injection, 61 (86%) showed a decrease in pain score to below 4. Of those, the pain score was 0 (none) in 28%, 1 (mild) in 28%, 2 (mild to moderate) in 6%, and 3 (moderate) in 38% (Figure 1; Figure 2). The median time to achieve pain control after the first injection was four months. Four months was also a common postoperative follow-up time for the practice. Therefore, four months was a common time interval during which data could be collected from the medical record. By 10 months, 85% of hip injections had a pain score below 4 at a clinic follow-up (Figure 3).

**Figure 4:**
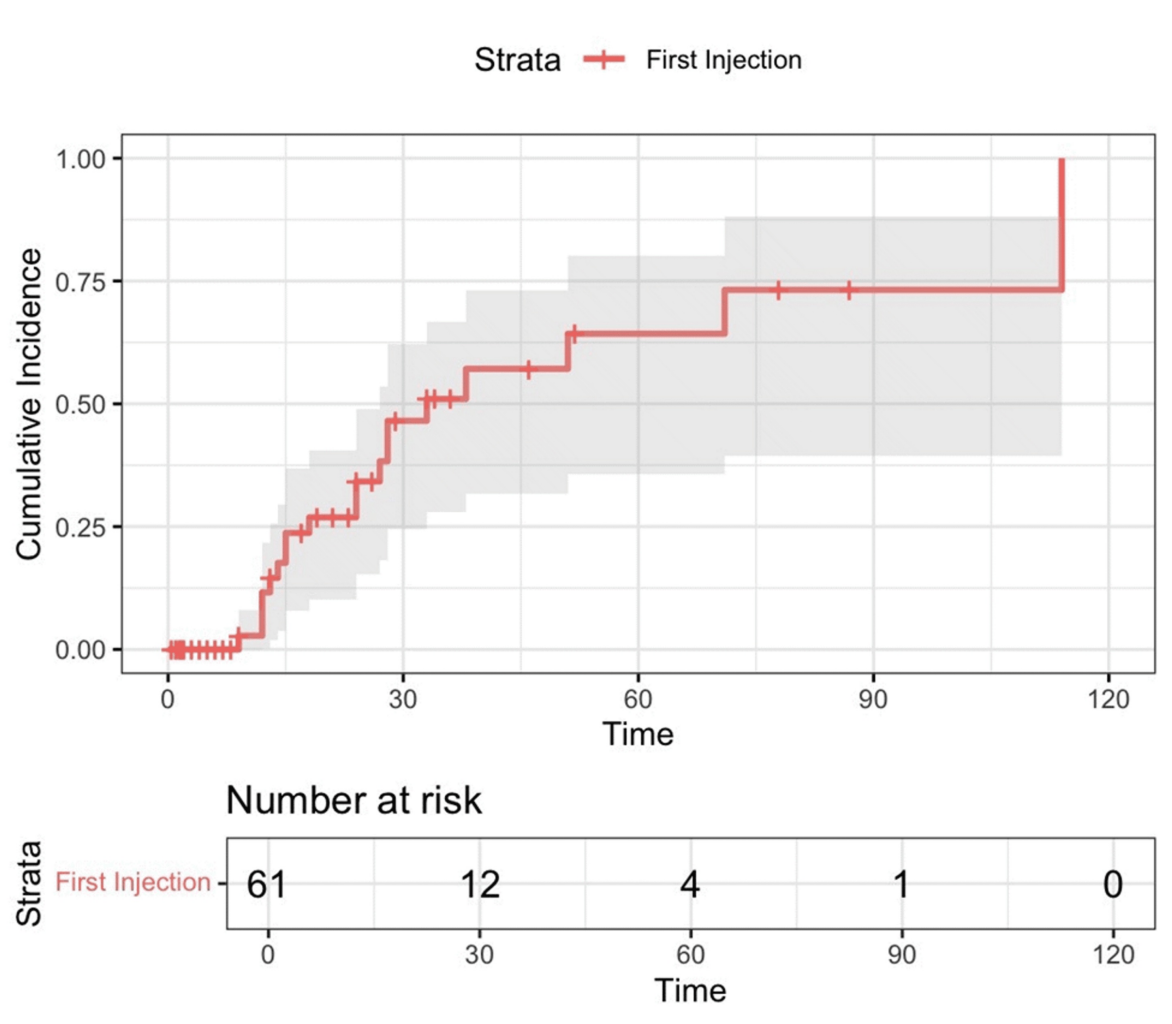
Kaplan-Meier cumulative incidence curve for time to second injection in patients who experienced pain control after the first injection. Time is in months. A cumulative incidence of 1.00 represents 19 hips. Of the 61 patient hips treated with the first ethanol injection who achieved pain control, 19 patient hips required a second injection. The median time to the second injection was 33 months.

Of the 61 hips that achieved adequate pain control, 19 (31%) required a second injection, with a median time to the second injection of 33 months. Among all 104 injections, 36% experienced pain relapse (Figure 4). The pain response seen following the first, second, third, and fourth injections varied between 80% and 100% (Table 2).

**Table 2:** Percentage of patients with efficacious pain management after each injection.

	Number of injections	% with efficacious pain management after injection
First injection	71	81%
Second injection	27	89%
Third injection	5	80%
Fourth injection	1	100%

In our population, following hip injection, 24 (34%) hips underwent hip reconstruction or resection surgeries, and six (8%) hips developed avascular necrosis of the femoral head, which was discovered incidentally on X-ray. Fisher’s exact test resulted in a P value of one, indicating no relationship between avascular necrosis and efficacious pain management after the first ethanol injection.

In addition to the intra-articular hip joint injections performed on 57 patients, most had additional procedures under the same anesthetic. They had the following minimally invasive procedures about the hip region: adductor tenotomies, hip flexor tenotomies, hamstring lengthening, obturator nerve ethanol blocks, and superior gluteal nerve ethanol blocks (Table 3).

**Table 3:** Minimally invasive procedures around the hip region under the same anesthetic. Number of patients: 57

Adductor tenotomy	Hip flexor lengthening	Hamstring lengthening	Obturator nerve block	Superior gluteal nerve block
46 (81%)	7 (12%)	29 (51%)	49 (86%)	3 (5%)

## Discussion

Building on the results of prior studies utilizing the injection of ethanol in various locations in the body, our study is the first to describe the clinical efficacy of ethanol blockade in CP patients when used as an intra-articular hip injection. These results are from a single center and are a single surgeon's outcomes. On average, these patients presented seeking an alternative to major salvage surgery.

Clinically and radiographically, these patients exhibited numerous preoperative characteristics. They had an average hip abduction value of -0.6 degrees (range, -35 to 35 degrees), indicating a mean severe adduction deformity. Additionally, the patients had a mean acetabular index of 33 degrees (range 10-90 degrees), indicating severe acetabular dysplasia, and femoral heads uncovered by a mean of 75% (range 0-100%), indicating a predominance of frankly dislocated hips. Hips with this degree of dysplasia qualify for salvage procedures based on X-ray appearance, yet many families favored a minimally invasive approach. Finally, in addition to intra-articular hip joint injections, most patients had additional procedures under the same anesthetic.

This study was not able to determine the relative pain-relieving effect of the hip joint injections versus the other procedures. Anecdotally, before the start of this study, the senior author used adductor tenotomy with obturator nerve block without hip joint injection for moderate-to-severe or severe hip pain in 10 patients, resulting in minimal pain reduction. A subsequent 10 patients who received the addition of ethanol hip joint injections experienced a noteworthy pain reduction.

The injection of ethanol within the hip capsule appears to provide temporary pain control for our patient population. The mechanism of action is unknown, but may be denaturing of intracapsular C-fiber fine nerve endings. Ethanol injection has been effective in controlling pain in various other locations in the body, as seen in chronic pain studies.

For our patient population of people with CP and a GMFCS of 4 or 5, an injection of 10 mL to 15 mL of 75% ethanol into the affected hip joint provided pain control in 86% of patients, with 31% having a second injection after a median of 33 months. The average follow-up period for the study was 43 months (range, 3-144 months), which was sufficient to document short-term and medium-term results.

There is concern for adverse events, such as avascular necrosis of the femoral head due to ethanol exposure to the intracapsular region and the development of neuritis, as observed in previous studies. In our patient group, six femoral heads (8%) developed avascular necrosis; whether this is related to the ethanol injection cannot be definitively stated, as it is known that patients with neurogenic dysplastic hips can develop avascular necrosis. The incidence of neuritis was seen in one patient (1%) and was transient, resolving after three months.

Ethanol injection provides an alternative to invasive hip reconstruction or salvage procedures in a patient population where pain control is the primary indication for treatment.

Limitations

This study has limitations in terms of generalizability, as it was conducted at a single center by a single surgeon in a retrospective chart review format. Additionally, the measurement of pain control was based on caregivers’ perceptions and patients’ facial expressions during physical exams in the clinic, which could introduce biases.

## Conclusions

This is the first study of an important modality. For our patient population with CP and a GMFCS level of 4 or 5, the intra-articular ethanol hip injection, when performed in addition to other minimally invasive procedures, has been shown to be beneficial in the medium term, providing effective pain control. This is a good option for patients with severe hip pain but with hips that are too dysplastic for reconstruction. In this study, there was an 8% incidence of avascular necrosis of the femoral head. Because of this, the injection treatment is most appropriate when bone resection salvage surgeries would otherwise be considered. Future studies need to be done to study the effect of different concentrations of ethanol on the incidence of avascular necrosis. Considering the evidence of efficacy presented regarding chronic pain treatment, further studies should be conducted to ascertain its use as a treatment modality. 
